# Antiviral activity of silver nanoparticle/chitosan composites against H1N1 influenza A virus

**DOI:** 10.1186/1556-276X-8-93

**Published:** 2013-02-20

**Authors:** Yasutaka Mori, Takeshi Ono, Yasushi Miyahira, Vinh Quang Nguyen, Takemi Matsui, Masayuki Ishihara

**Affiliations:** 1 Third Division, Aeromedical Laboratory, Japan Air Self-Defense Force, 2-3 Inariyama, Sayama, Saitama, 350-1324, Japan; 2 Research Institute, National Defense Medical College, 3-2 Namiki, Tokorozawa, Saitama, 359-8513, Japan; 3 Department of Global infectious Diseases and Tropical Medicine, National Defense Medical College, 3-2 Namiki, Tokorozawa, Saitama, 359-8513, Japan; 4 Faculty of System Design, Tokyo Metropolitan University, 6-6 Asahigaoka, Hino-shi, Tokyo, 191-0065, Japan

**Keywords:** Organic/metal nanocomposites, Biomass materials, Antimicrobial materials, Polysaccharides, Nanotoxicity

## Abstract

Silver nanoparticle (Ag NP)/chitosan (Ch) composites with antiviral activity against H1N1 influenza A virus were prepared. The Ag NP/Ch composites were obtained as yellow or brown floc-like powders following reaction at room temperature in aqueous medium. Ag NPs (3.5, 6.5, and 12.9 nm average diameters) were embedded into the chitosan matrix without aggregation or size alternation. The antiviral activity of the Ag NP/Ch composites was evaluated by comparing the TCID_50_ ratio of viral suspensions treated with the composites to untreated suspensions. For all sizes of Ag NPs tested, antiviral activity against H1N1 influenza A virus increased as the concentration of Ag NPs increased; chitosan alone exhibited no antiviral activity. Size dependence of the Ag NPs on antiviral activity was also observed: antiviral activity was generally stronger with smaller Ag NPs in the composites. These results indicate that Ag NP/Ch composites interacting with viruses exhibit antiviral activity.

## Background

Silver nanoparticles (Ag NPs) are well-known antimicrobial materials effective against many types of bacteria [1-3] and fungi [4]. The antibacterial and antifungal activities of Ag NPs are mainly due to the inhibition of respiratory enzymes by released Ag^+^ ions [1,5]. Recently, the antimicrobial activities of Ag NPs against viruses such as HIV-1 [6,7], hepatitis B [8], herpes simplex [9], respiratory syncytial [10], monkeypox [11], Tacaribe [12], and H1N1 influenza A virus [13,14] have also been investigated. Unlike its antibacterial and antifungal activities, the major antiviral mechanism of Ag NPs is likely the physical inhibition of binding between the virus and host cell. A dependence of the size of Ag NPs on antiviral activity was observed for the viruses mentioned above; for example, Ag NPs smaller than 10 nm specifically inhibited infection by HIV-1 [6]. This property of Ag NPs holds promise that antimicrobial materials based on Ag NPs will be effective against many types of bacteria, fungi, and viruses.

On the other hand, there are some concerns about the biological and environmental risks of Ag NPs. It is known that Ag NPs have adverse effects, such as cytotoxicity and genotoxicity on aquatic organisms like fish [15], and can inhibit photosynthesis in algae [16]. One study on mammals showed a significant decline in mouse spermatogonial stem cells following the administration of Ag NPs [17]. Therefore, preventing the diffusion and intake of Ag NPs into the environment and the biosphere are important considerations in the design of antimicrobial materials containing Ag NPs [18-22]. One approach would be the fixation of Ag NPs into matrices; for example, Fayaz et al. have prepared Ag NP-coated polyurethane and have demonstrated its antiviral activity against HIV-1 and herpes simplex virus [23]. Nevertheless, the efficacy and mechanism of action of such Ag NP-fixed antiviral materials against various viral strains are not well investigated.

In this paper, the antiviral activity of Ag NP/polymer composites against H1N1 influenza A virus was investigated. Chitosan (Ch), which is the main constituent of the exoskeleton of crustaceans and exhibits strong antibacterial activity [24], was used as the matrix polymer. Controlling the size of Ag NPs is as important to antiviral activity as the composition of the Ag NPs. We previously demonstrated an environmentally friendly process for producing Ag NPs with a narrow size distribution [25]. This process uses only three materials: a silver-containing glass powder as an Ag^+^ supplier, glucose as a reducing agent for Ag^+^, and water as a solvent. The stabilizing agent for Ag NPs is caramel, which is generated from glucose during heating to reduce Ag^+^. In this work, Ag NPs synthesized by this process were used to make the Ag NP/Ch composites, since the size of the Ag NPs could be easily controlled without the use or production of hazardous materials. Ag NP/Ch composites were synthesized in aqueous media at room temperature by mixing a chitosan solution and an Ag NP suspension. The surface and internal structure of the synthesized Ag NP/Ch composites were observed by scanning and transmission electron microscopies, respectively. The effect of introducing a small amount of Ag NPs into the chitosan matrices and the effect of the size of the Ag NPs were evaluated with respect to the antiviral activity of the composites.

## Methods

### Materials

Ag NP suspensions were synthesized from silver-containing glass powder (BSP21, silver content 1 wt%, average grain size 10 μm, Kankyo Science, Kyoto, Japan) and glucose aqueous solution, as described previously [25]. Ag NPs used in this work were spherical; their characteristics are summarized in Table 1. Phosphate-buffered saline (PBS), methanol, Giemsa stain solution, and 5 M hydrochloric acid (HCl) and 5 M sodium hydroxide (NaOH) aqueous solutions were purchased from Wako Pure Chemical Industries, Ltd. (Osaka, Japan) and used without further purification. Chitosan solution (10 mg/mL) was prepared by mixing 0.1 g chitosan (average molecular weight 54 kg/mol, deacetylation ratio 84%; Yaizu Suisankagaku Industry Co., Ltd., Shizuoka, Japan), 10 mL of PBS, and 100 μL of 5 M HCl; following complete dissolution of the chitosan, the solution was filter-sterilized by passage through a 0.2-μm filter. Bovine serum albumin (BSA) solution was prepared using BSA powder (Sigma-Aldrich Japan, Tokyo, Japan) and PBS, then filter-sterilized as above. Trypsin was obtained from Life Technologies Co., (Carlsbad, CA, USA). Dulbecco's Modified Eagle Medium (DMEM, high glucose) was purchased from Sigma-Aldrich Japan (Tokyo, Japan).

**Table 1:** Characteristics of Ag NPs

**Sample number**	**Average diameter ± SD (nm)**	**Concentration of Ag NP in suspension (μg/mL)**
SN35	3.5 ± 1.8	73
SN65	6.5 ± 1.8	62
SN129	12.9 ± 2.5	77

### Synthesis of Ag NP/Ch composites

Chitosan solution (100 μL, 10 mg/mL) was mixed with Ag NP solution (0.25 to 4.5 mL) and 40 μL 5 M NaOH at room temperature, followed by vigorous stirring to precipitate the Ag NP/Ch composite. The obtained Ag NP/Ch composite was centrifuged at 6,000 rpm for 10 min. The supernatant was analyzed using a UV-visible spectrometer (JASCO V-630, Tokyo, Japan) to estimate the amount of unreacted Ag NPs. Centrifuged composites were washed with 1 mL PBS, followed by centrifugation at 6,000 rpm for 10 min. The washing process was repeated twice. The washed Ag NP/Ch composite was suspended in 250 μL PBS and used in antiviral assays the same day. Synthesis of the Ag NP/Ch composites was carried out in a laminar flow cabinet to prevent biological contamination.

### Microscopy observations

Scanning electron microscopy (SEM) specimens of the composites were prepared by casting 5 μL of a water dispersion of the Ag NP/Ch composite, followed by drying at room temperature. Osmium plasma coating was conducted to enhance the conductivity of the specimens. Dried samples were coated using a plasma multi-coater PMC-5000 (Meiwafosis Co., Ltd., Tokyo, Japan). SEM observation was performed using a JSM-6340F (JEOL, Tokyo, Japan) at 5 kV. Transmission electron microscopy (TEM) specimens of the Ag NPs and Ag NP composites were prepared by casting 5 μL of Ag NP solution or a water dispersion of the composite onto a carbon-coated copper microgrid. Excess solution was removed using filter paper, and the specimens were dried at room temperature. Further staining was not carried out for any specimen. TEM observation was performed using a JEM-1010 (JEOL) at 80 kV.

### Assaying the antiviral activity of the Ag NP/Ch composites

Human influenza A virus (A/PR/8/34 (H1N1)), obtained from Life Technologies Co., was used and assayed using the fifty-percent tissue culture infectious dose (TCID_50_) method. Viral suspension in PBS (250 μL, titer ca. 1,000 TCID_50_/mL) was added to 250 μL Ag NP/Ch composite suspension. The mixture was stirred vigorously for 5 s and then left at room temperature for 1 h to allow the virus and composite particles to interact. Then, the mixture was centrifuged at 6,000 rpm for 10 min to remove the composite particles. The supernatant (50 μL) was subjected to two-fold serial dilution with PBS 11 times in a 96-well cell culture plate sown with Madin-Darby canine kidney (MDCK) cells. Eight duplicate dilution series were prepared and assayed for each Ag NP/Ch sample. Samples were incubated at 37°C and 5% CO_2_ for 1 h to allow viral infection of the MDCK cells. MDCK cells were maintained by adding 50 μL DMEM (with the addition of 0.4% of BSA and 5 ppm of trypsin) to each well immediately following infection and again 5 days post-infection. Seven days post-infection, the living cells were fixed with methanol and stained with 5% Giemsa stain solution. The TCID_50_ of the sample solution was calculated from the number of infected wells using the Reed-Muench method [26,27]. The antiviral activity of the Ag NP/Ch composite was estimated as the TCID_50_ ratio of the Ag NP/Ch-treated supernatant to the control (untreated) viral suspension.

## Results and discussion

**Figure 1:**
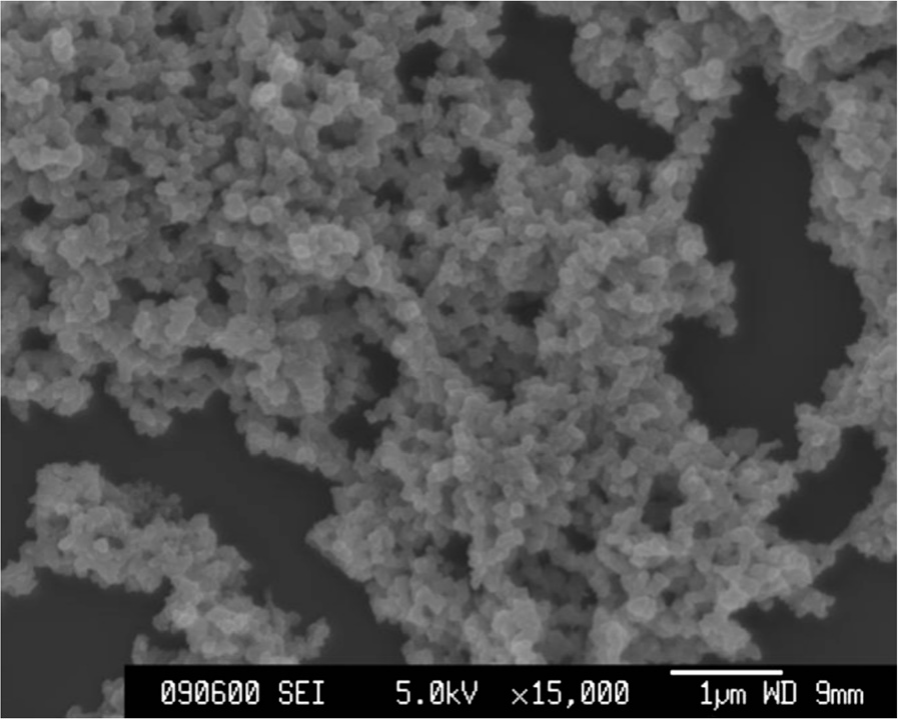
**A SEM micrograph of chitosan/SN129.** Weight ratio of Ag NPs in the composite is 23.5 wt%.

**Figure 2:**
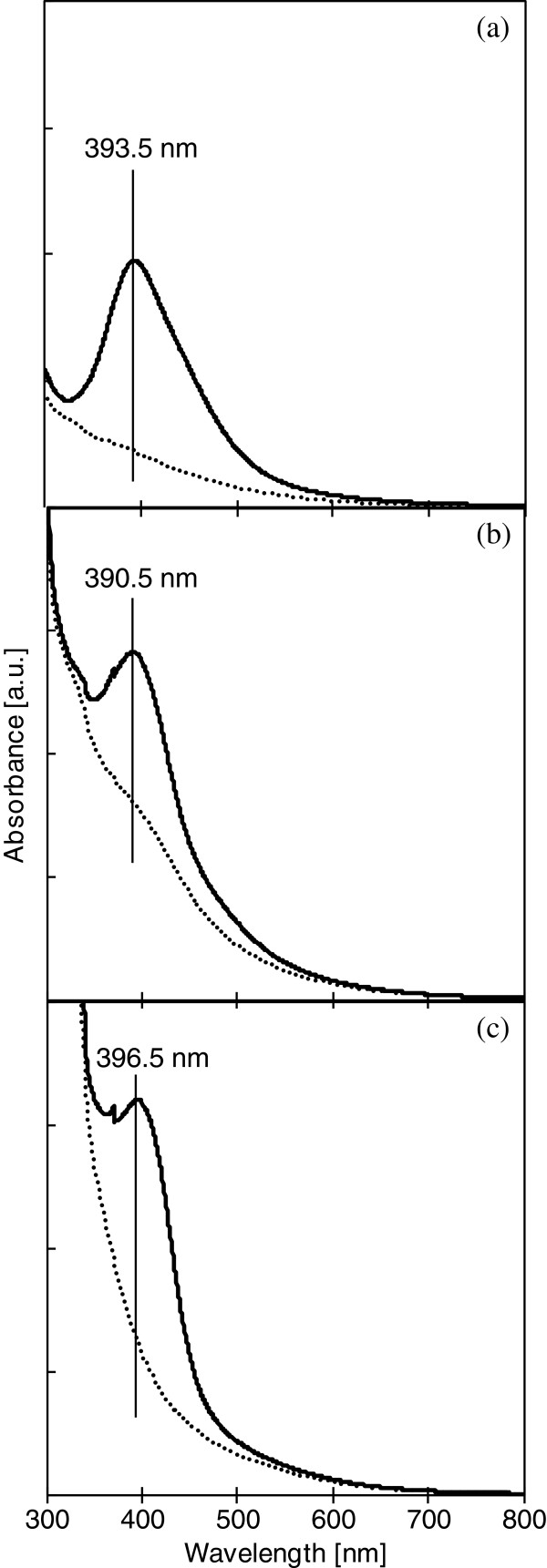
**UV-visible spectra of the original Ag NP suspension and of the post-reaction mixture supernatant.** Solid line and dashed line correspond to the original Ag NP suspension and the post-reaction mixture supernatant, respectively. (**a**) SN35 and the supernatants obtained from 1 mg of chitosan and 328.5 μg of SN35, (**b**) SN65 and the supernatants obtained from 1 mg of chitosan and 279 g μof SN65, (**c**) SN129 and the supernatants obtained from 1 mg of chitosan and 308 μg of SN129. The peak due to Ag NPs is marked with a vertical line. The supernatants were obtained from the post-reaction mixture of 1 mg of chitosan and 328.5 μg of SN35 (dotted line), 279 μg of SN65 (short dashed line), and 308 μg of SN129 (long dashed line). The solid line corresponds to the original suspension of SN129.

Ag NP/Ch composites were synthesized by mixing a chitosan acidic aqueous solution with an Ag NP suspension. Chitosan is water soluble in acidic conditions due to protonation of primary amines in the chitosan chains. The Ag NP suspension was also acidic (pH 5.23 to 6.25) [25]. Although the acidity of these two solutions was maintained during mixing, partial precipitation of the Ag NP/Ch composites was observed at all conditions tested, suggesting that decreased solubility of the chitosan chains was induced by the binding of Ag NPs to the chitosan amino and hydroxyl groups [28]. Addition of excess NaOH completely precipitated the composite. Figure 1 shows a typical SEM micrograph of the composite. Ag NP/Ch composites were obtained as flocculated, aggregated, spherical sub-micrometer particles. The composites were yellow or brown; darker composites were obtained when larger amounts of Ag NPs were reacted with the chitosan. Figure 2 shows UV-visible spectra of the original Ag NP suspension and of the reaction mixes containing high amounts of Ag NP. Since spherical Ag NPs provide a peak near 400 nm [25,29], the absence of this peak shows that Ag NPs are not present in the supernatant of the post-reaction mixture and that the Ag NPs were completely bound to the chitosan.

**Figure 3:**
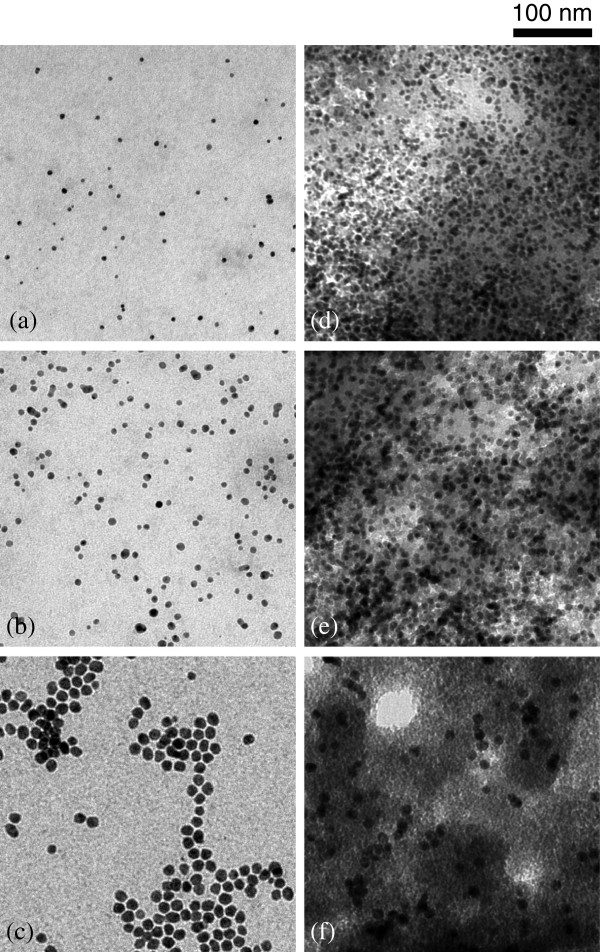
**TEM micrographs of Ag NPs.** (**a**) SN35, (**b**) SN65, (**c**) SN129; Ag NP/Ch composites (**d**) 24.7 wt% of SN35, (**e**) 21.8 wt% of SN65, (**f**) 23.5 wt% of SN129.

TEM micrographs of the Ag NPs and Ag NP/Ch composites are shown in Figure 3. Compared to Ag NPs before reaction, Ag NPs in the composites are dispersed in the chitosan matrix and appear as uneven gray domains. The thickness of the TEM specimen of the composites is uneven due to the direct casting of the composite floc. Uneven contrast of the chitosan domains is due to the uneven thickness of the specimen. Ag NPs in thick areas of the chitosan matrix are overlapped. Meanwhile, Ag NPs in thin areas appeared non-overlapped. The particle sizes of Ag NPs in the composites are similar to that of the original Ag NPs. Although some minor aggregation of Ag NPs was observed, there was no macroscopic aggregation, showing that the particle size of the Ag NPs in the Ag NP/Ch composites was controlled.

**Figure 4:**
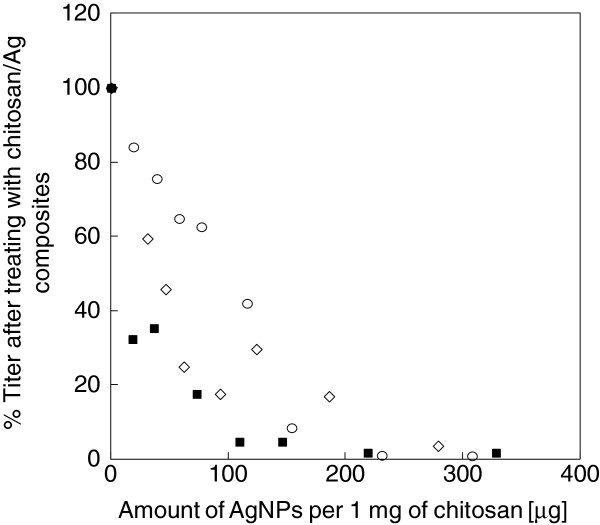
**Relationship between the anti-influenza virus activity of Ag NP/Ch composites and their composition.** SN35 (square), SN65 (diamond), and SN129 (circle).

Figure 4 shows the dependence of particle size and amount of Ag NPs on the antiviral activity of the composites against influenza A virus. The TCID_50_ ratios of viral suspensions treated with Ag NPs and Ag NP/Ch composites to untreated suspensions were used to gauge the antiviral activity of the materials. For all Ag NPs tested, the antiviral activity of the Ag NP/Ch composites increased with increasing amount of Ag NPs. No antiviral activity was observed with chitosan alone, showing that the antiviral activity of the composites was due to the bound Ag NPs. The effect of size of the Ag NPs in the composites was also observed: for similar concentrations of Ag NPs, stronger antiviral activity was generally observed with composites containing smaller Ag NPs. This size effect was most prominent when less than 100 μg of Ag NPs was added to 1 mg of chitosan. No increase in antiviral activity was observed above 200 μg of Ag NPs per 1 mg of chitosan, irrespective of the size of the Ag NPs.

Previous studies showed that Ag NPs have antiviral activity against influenza A virus [13,14]. Although the mechanism of action has not been well investigated, it is likely that the antiviral activity of Ag NPs against several other types of viruses is due to direct binding of the Ag NPs to viral envelope glycoproteins, thereby inhibiting viral penetration into the host cell [6,8,13,30]. The effect of the size of Ag NPs on antiviral activity was usually observed, suggesting spatial restriction of binding between virions and Ag NPs [6,8]. For the Ag NP/Ch composites, further spatial restriction due to the chitosan matrix would be expected to prevent or weaken the interaction between virions and Ag NPs. On the other hand, physical binding of virions to the composites could directly inhibit viral contact with host cells since the virus-treated composites were removed from the assay solution prior to infection of the host cells. When embedded Ag NPs could interact with the virions, the interaction between the virions and the composites should increase with increased concentration of Ag NPs in the composites; this is supported by the experimental results on the relationship between the antiviral activity and the concentration of Ag NPs. The effect of the size of Ag NPs in the composites on antiviral activity suggests that influenza A virus interacted selectively with smaller Ag NPs, as previously reported for other types of viruses [6,8]. However, the size dependence of free Ag NPs on antiviral activity against influenza A virus has not been studied. To obtain more effective Ag NP-embedded antiviral materials, detailed studies of the mechanism of antiviral action of both free and embedded Ag NPs are required. The effects of the microscopic structure and the properties of Ag NP-embedded materials on antiviral activity should also be investigated in the future. Nonetheless, this study clearly demonstrates the feasibility of using Ag NPs to impart antiviral activity to chitosan and lower concerns about the risk of diffusion of Ag NPs in the environment.

## Conclusions

Ag NP/Ch composites with antiviral activity against influenza A virus were synthesized in aqueous medium. The composites were obtained as yellow or brown flocs; unreacted Ag NPs were not detected in the residual solution. The particle size of the Ag NPs in the composites was similar to that of the Ag NPs used to synthesize the composites. The antiviral activity of the composites was determined from the decreased TCID_50_ ratio of viral suspensions after treatment with the composites. For all sizes of Ag NPs tested, the antiviral activity of the Ag NP/Ch composites increased as the amount of Ag NPs increased. Stronger antiviral activity was generally observed with composites containing smaller Ag NPs for comparable concentrations of Ag NPs. Neat chitosan did not exhibit antiviral activity, suggesting that Ag NPs are essential for the antiviral activity of the composites. Although the antiviral mechanism of the composites remains to be investigated, the experimental results showing the relationship between antiviral activity and the concentration of Ag NPs suggest that the virions and composites interacted. Consequently, detailed studies of the antiviral mechanism of the Ag NP/Ch composites could lead to the development of practical Ag NP-containing materials that will reduce concerns about the risks of diffusion of Ag NPs into the environment.

## Abbreviations

Ag NP: Silver nanoparticle; Ch: Chitosan; SEM: Scanning electron microscopy; TEM: Transmission electron microscopy; TCID_50_: Fifty-percent tissue culture infectious dose.

## Competing interests

The authors declare that they have no competing interests.

## Authors' contributions

YMo designed the research, performed the experiments, and drafted the manuscript and the figures. TO guided and performed the viral study. YMi supervised the virus study. VQN performed some of the experiments. TM participated in the design of the research. MI supervised and coordinated the study and approved the manuscript. All authors read and approved the final manuscript.

## Authors' information

YMo is a technical official of the Japan Air Self-Defense Force. MI and YMi are professors of the National Defense Medical College. TO is a research associate of the National Defense Medical College. TM is a professor of the Tokyo Metropolitan University. VQN is a graduate student of the Tokyo Metropolitan University.
